# The Physiological Anatomy and Physiology of Man

**Published:** 1857-10

**Authors:** 


### BOOK NOTICES.

*The Physiological Anatomy and Physiology of Man. By Robert Bently Todd, M. D., F. R. S., etc., etc., and William Bowman, F. R. S., etc., etc. Complete in one volume, with two hundred and ninety-eight illustrations. Blanchard and Lea, Philadelphia. Keen and Lee, Chicago,* 1857.

The authors began the arduous and difficult task of writing the above work in the year 1843, intending to have issued it much sooner, and to have made it only subserve the purpose of a text book for the Lectures on General Anatomy and Physiology, given in King's College, London. Finding the sciences of Anatomy and Physiology becoming daily more and more extended, the field for observation and study rapidly enlarging, and many new subjects presenting themselves, requiring further and more careful investigation, they were compelled to delay its publication thus long, hoping the additions and modifications it enabled them to make would compensate for the procrastination. No one acquainted with the science of medicine but that feels within himself the very prominent position Anatomy and Physiology hold therein. They are the groundworks of our professional knowledge, and the great barrier that separates us from the imposing charlatan. The student will find them indispensible to the successful prosecution of his studies; the physician, invaluable interpreters at the bed-side. This branch of our science, then, so important, could not well escape the notice of our most eminent men, whose enthusiasm has led to investigation and study so thorough as to transform it almost into a new being. Nothing seems better calculated to stimulate us to greater zeal and exertion than the advances made in this department of our science within the last few years. The above work shows how patiently and how sedulously its authors must have labored to achieve their part in the promotion of these sciences, and how well they have succeeded. Its perusal will at once test its superior merit, and in no other way can our friends expect to become acquainted with its contents. We do not intend to write a critical analysis of the work, but merely a brief notice of its contents, sufficient to enable our readers to judge of its extent and importance. It is a large-sized volume, containing over 900 pages, is plainly printed, and handsomely bound. Besides the introduction it contains 44 chapters, which comprise the consideration of the following subjects: i, Of the Constituents of Animal Bodies—The Tissues and their Properties; ii, Of the Minute Movements occurring in the Body; iii, iv, v, vi, Of Locomotion—Its Passive Organs; vii, Active Organs of Locomotion; viii, ix, x, xi, xii, xiii, xiv, xv, xvi, xvii, xviii, xix, xx, xxi, Of Innervation; xxii, xxiii, xxiv, xxv, Digestive; xxvi, Absorption; xxvii, The Blood; xxviii, The Circulation of the Blood; xxix, Respiration; xxx, Animal Heat; xxxi, The Voice; xxxii, Secretion; xxxiii, xxxiv, Secreting Glands; xxxv, Ductless Glands; xxxvi, xxxvii, xxxviii, xxxix, xl, Generation; xli, xlii, xliii, Development; xliv, Lactation. Having thus enumerated the headings of each chapter, our readers at once perceive the vast amount of matter which this volume brings before us. Questions of difficult solution, and such as yet seem to be unsettled, are all discussed and disposed of with apparent satisfaction to our authors, and doubtless with as much correctness as the present state of our knowledge will admit. That we are yet far from well ascertained and incontestable proof as regards the functions of life, few will hesitate to deny. In the introduction, our authors, in discussing the different "theories of life," have placed the subject before us on physical and chemical principles quite prominently. They say: "Many of the phenomena of life may be accounted for on chemical or physical principles. The changes effected in the air and in the blood by respiration, the phenomena of absorption, and, in some degree, those of secretion, are the results of purely physical processes." They also suggest the probability that the actions of the nervous system are due to physical changes, digestion to a chemical process, and the generation of heat to the same chemical phenomena as will give rise to it in the inorganic world. Aristotle and Harvey attributed the organization of animals and vegetables, and the vital actions exhibited by them to a series of "Animating Principles." Muller, to the presence of an "Organic Force." Prout, to certain "Organic Agents." Hunter, to a "Materia Vitæ." Each of these theories have, for a time, sustained a reputation of correctness, and were looked upon as being able to account for that principle we call "Life," but have each, in turn, been found wanting. The chemical and physical explanation was long since adopted, and has had many able advocates who still support it, with at least a plausibility quite equal to the other, and, at present, its most formidable rival, the "vital principle." Time and research must settle this question. Further on in the introduction we find a statement which has attracted our attention, more particularly as it is so directly opposed to the doctrine of M. Bernard of Paris, who has recently written a work on Physiology. M. Bernard may perhaps be fairly considered as the greatest experimenter on the living or recently killed animals in the world, and his experiments have inaugurated a new epoch in the science of Physiology. He adopts an entirely different method of investigation from that of any previous author, and one quite opposed to that recommended by Todd and Bowman. M. Bernard starts with the function or physiological phenomena, and endeavors to trace it back to the organ which gives rise to it. He does not believe we can, by first making ourselves acquainted with the anatomy of an organ, determine its use, whilst our authors say: "The study of Anatomy must always accompany that of Physiology, on the principle that we must understand the construction of a machine before we can comprehend the way in which it works." Starting, then, with entirely different views as regards the proper mode of studying this branch of our science, we may reasonably suppose they often differed in their results or conclusions. In Chapter XI. we find our authors, in speaking of the "Functions of the Spinal Cord," attribute it to a "polar state," capable of producing actions quite independent of the will or sensation. This "polarity" may be produced either by direct irritation of its substance, or by the influence of a stimulus conveyed to it through the medium of the nervous trunks. Tetanus is given as an example of this form of action. Opium is also capable of producing it, and is, consequently, inadmissible in that disease. Epilepsy is explained on the same hypothesis. This class of actions they attribute entirely to physical changes produced in the nervous centres and trunks.

The celebrated theory of Gall respecting the functions of the cerebellum they deem far from admissible, and have supported their argument on quite reasonable, if not conclusive grounds. They adopt the conclusion drawn by Flourens, "that the cerebellum possesses the power of coordinating the voluntary movements which originate in other parts of the cerebro-spinal centre, whether these movements have reference to locomotion or to other objects."

In concluding this chapter the following inferences are set forth.

I. "The Spinal Cord contains within itself all the physical conditions necessary for the mental and physical actions of the trunk and extremities, so long as its connection with the encephalon is perfect through the anterior pyramids.

II. "There is no sufficient evidence to prove the existence of a class of sensori-volitional fibres distinct from those which are the instruments of physical actions.

III. "Each segment of the cerebro-spinal centre, whether in the cranium or in the spinal canal, gives origin to its own proper nerves, and has no connection with the neighbouring segments otherwise than by commisural fibres or vesicular matter.

IV. "The antero-lateral columns of the cord, with the anterior and posterior horns of the gray matter, are the effective centres of motion and sensation of the trunk and extremities. The posterior columns are longitudinal commissures, by which the influence of the cerebellum is brought to bear on the various segments of the cord.

V. "When the pyramids are in a state of integrity the corpus striatum, certain accumulations of gray matter connected with the nerves of the medulla oblongata, the locus niger, and the anterior horns of the spinal gray matter, are the centres of voluntary motion to the whole body ; while the optic thalami, olivary columns and posterior horns of gray matter are the centres of sensation.

VI. "The medulla oblongata, when connected to the corpora striata by the pyramidal fibres, is a centre of voluntary actions to those parts whose nerves are derived from it; and, in addition, it is the principle centre of the actions of respiration and deglution.

VII. "The corpora quadrigemina are primary centres of visual impressions, and, with a large portion of the gray matter in the mesocephale, are centres of emotional actions.

VIII, "The cerebellum is the co-ordinator of voluntary and locomotive actions.

IX. "The convolutions of the brain are the centres of intellectual actions, and are intimately associated with the mental phenomena of attention, association and memory."

After considering briefly the subject of sympathetic actions Todd and Bowman have taken up in regular order the special senses. Beginning with that of touch, they first give the minute anatomy of the skin and its appendages, then define it as it becomes the "*organ of touch*," and, finally, describe the peculiarity of different impressions, their seat, and duration. The same order is observed in studying the sense of taste, smell, hearing and vision. Each is preceded by a full description of all the parts concerned in their production, their exact location determined, and those agents which most influence them. The whole subject has received from them special care, and is studied with great satisfaction and profit. Chapter xix treats of those nerves exclusively motor in function. To this class belong the third, fourth, sixth, seventh and ninth pairs. The fifth and eighth pairs are compound in their operations. The fifth is one of the most interesting and extensively connected in the whole body. The eighth pair consists of three separate nerves, the *pneumogastric*, the *glossopharyngeal*, and the *spinal*

*accessory*. A detailed account of each is given, and their physiological action. The following conclusions are drawn respecting the pneumogastric and its branches :

1. "That the vagus nerve contains filaments both of sensation and motion.

2. "That its pharyngeal branches are motor.

3. "That its superior laryngeal branch is the sensitive nerve of the larynx, containing a few motor filaments to the crico-thyroid."

4. "That the inferior laryngeal is the principal motor nerve of the larynx."

5. "That the cardiac branches exert a slight influence on the movements of the heart."

5. "That its pulmonary branches contain both motor and sensitive filaments, and exercise an important influence on the respiratory acts, for they cannot be destroyed without retarding materially the respirations, impeding the passage of blood through the lungs, and causing ordema of these organs."

7. "That its oesophageal branches are the channels through which the muscles of that tube are excited, through the medulla oblongata, to contract."

8. "That the gastric branches influence the movements of the stomach, and, probably, in some degree the secretions and the sensibility of its mucus membrane; but that their integrity is by no means essential to the continuance of this secretion, or to complete chymification."

Four chapters are devoted to the study of digestion. Our authors first classify the different kinds of food according to Prout. They then give the anatomy of the several organs concerned in this process, and the function of each. The saliva, the gastric juice, the bile, and the pancreatic juice, are each studied separately, and the parts they play in this organic function clearly assigned. The glands of the intestines and their secretions are quite elaborately considered, and many interesting experiments detailed. The next subject in order, is absorption ; explained on purely chemical and physical principles. Following this, the examination of the blood; *first*, as regards its quantity; *second*, its coagulability; *third*, its physical analysis; and *fourth*, its chemical analysis. Its circulation comprises the subject of one chapter, in which is given the minute anatomy of the arteries, veins, capillaries, and the central organ of circulation, to precede the study of the influence exerted by each in propelling their contents. Respiration, and animal heat are next studied in connection with the changes effected in the air and blood by the former, and also the changes going on in the animal economy, giving rise to the production of the latter. The consideration of the "ductless glands" is taken up in chapter xxxv., and after entering minutely into the anatomy of their substance, their bloodvessels, nerves and lymphatics, they proceed to point out the functions they perform. To the spleen, they assign the office first advanced by Holliker, in 1847, and afterwards supported by Ecker and Beclard, viz: "the disintrigation of blood corpuscles." This theory is directly opposed to those who have adopted the view that blood corpuscles were formed in the spleen. As regards the functions of the supra-renal capsules, the thyroid and thymus glands nothing is definitely known. The work closes with the study of Generation and Lactation. The interesting and intricate subject of generation is fully treated, and contains much valuable instruction. In it we find much that would afford questions of controversy, and difference of opinion, as. perhaps, we ever shall, on all subjects so mysterious and obscure.

Our notice, though it conveys but a very feeble and imperfect idea of the magnitude and importance of the work now under consideration, already transcends our proper limits; and, with the indulgence of our readers, and the hope that they will peruse the book for themselves, as we feel we can with confidence recommend it, we leave it in their hands, for them to judge of its merits. G. K. A.

